# Creating an Optimality Index – Netherlands: a validation study

**DOI:** 10.1186/s12884-018-1735-z

**Published:** 2018-04-16

**Authors:** Suzanne M. Thompson, Marianne J. Nieuwenhuijze, Luc Budé, Raymond de Vries, Lisa Kane Low

**Affiliations:** 1 Research Centre for Midwifery Science Maastricht, Zuyd University, Universiteitssingel 60, 6229ER Maastricht, The Netherlands; 2 Care and Public Health Research Institute (CAPHRI), Maastricht University, Maastricht, The Netherlands; 3 University of Michigan, Ann Arbor, USA

**Keywords:** The Netherlands, Physiological childbirth, Optimality, Validation, Optimality Index-Netherlands

## Abstract

**Background:**

At present, the maternity care system in the Netherlands is being reorganized into an integrated model of care, shifting the focus of midwives to include increasing numbers of births in hospital settings and clients with medium risk profiles. In light of these changes, it is useful for midwives to have a tool which may help them in reflecting upon care practices that promote physiological childbirth practices. The Optimality Index-US is an evidence based tool, designed to measure optimal perinatal care processes and outcomes. It has been validated for use in the United States (OI-US), United Kingdom (OI-UK) and Turkey (OI-TR). The objective of this study was to adapt the OI-US for the Dutch maternity care setting (OI-NL).

**Methods:**

Translation and back translation were applied to create the OI-NL. A panel of maternity care experts (*n* = 10) provided input for face validation items in the OI-NL. Assessment of inter-rater reliability and ease of use was also conducted. Following this, the OI-NL was used prospectively to collect data on 266 women who commenced intrapartum care under the responsibility of a midwife. Twice groups were compared, based on parity and on care-setting at birth. Mean scores between these groups, corrected for perinatal background factors were assessed for discriminant validity.

**Results:**

Face validity was established for OI-NL on the basis of expert input. Discriminant validity was confirmed by conducting multiple regressions analyses for parity (β = 6.21, *P* = 0.00) and for care-setting (β = 12.1, *p* = 0.00). Inter-rater reliability was 98%, with one item (Apgar score) sensitive to scoring differences.

**Conclusion:**

OI-NL is a valid and reliable tool for use in the Dutch maternity care setting. In addition to its value for assessing evidence-based maternity care processes and outcomes, there is potential for use for learning and reflection. Against the backdrop of a changing maternity care system, and due to the specificity of its items OI-NL may be of value as a tool for detecting subtle changes indicative of escalating medicalization of childbirth in the Netherlands.

**Electronic supplementary material:**

The online version of this article (10.1186/s12884-018-1735-z) contains supplementary material, which is available to authorized users.

## Background

In the Netherlands, midwives in primary care are responsible for care provision to healthy women with uncomplicated pregnancies. Women are referred to obstetrician-led care (secondary care) by midwives when there are complications or an increased risk of complications. Increasingly, midwives are also working in hospitals under the supervision of obstetricians, where they care for the majority of women who have been referred because of complications in pregnancy or birth [1, 2].

A physiological approach to childbirth is considered a core midwifery competency [3] and Dutch midwives report viewing the promotion and support of physiological childbirth as fundamental to their role [4]. However, this viewpoint appears at odds with quantitative studies from the Netherlands that demonstrate increasing numbers of non-urgent referrals to obstetric-led care in the intrapartum period and a broad diversity in referral rates between midwifery practices, varying between 9.7 and 63.7% [5]. It is unlikely that these differences are caused by risk of complications because of different population characteristics alone, but could also be related to differences in the ways midwives practice or to midwife perceptions of the likelihood of adverse events in birth [6, 7]. Moreover, variations in specific areas of midwifery practice have been noted, including a high variation in the incidence of episiotomy between Dutch primary care midwifery practices [8]. Another example of a less physiological approach to childbirth is the limited use of non-supine birthing positions in hospital and, notably, primary care settings [9].

These examples suggest that Dutch midwives sometimes find it challenging to promote and support physiological childbirth. Hospital settings may negatively impact on the ways in which midwives facilitate physiological childbirth [10]. Other challenges to physiological childbirth practice may include environmental factors such as medical hierarchy or nursing staff. Hospital midwives may feel inhibited in their role as promoters and supporters of physiological childbirth in the hospital setting, which is oriented towards a biomedical, rather than a holistic approach to childbirth. This is of concern as physiological childbirth, a complex biological and hormonal process [11] which unfolds naturally when undisturbed [12], can be empowering [13] and salutogenic [14]. While obstetric interventions are beneficial in certain situations, the over-use of these can result in unintended consequences, including iatrogenic harm [15] to individual women and their babies and unsustainable economic costs for the wider society [16].

At present, the Dutch midwifery system is evolving towards an integrated model of care, and increasing numbers of births take place in hospital settings. Integrated care includes an extended remit for midwives to provide care both medium risk and low risk women. Rates of obstetric interventions in the Netherlands remain still low compared to other industrialized countries [17], however, there is concern among midwives and others that the change to an integrated model of care may lead to more interventions [18, 19].

Against the backdrop of significant changes to the Dutch model of midwifery, it is helpful to have a tool that can be used to investigate developments in the intrapartum use of interventions and promote reflection on the evidence-based use of these interventions, and thereby prevent the escalation towards unnecessary disruption of physiological childbirth. One such tool is the Optimality Index (OI).

### Optimality and the Optimality Index

Optimality as a concept in perinatal health care, based on work done by Prechtl [20], can be defined as the maximal perinatal outcome, with minimal intervention, placed within the context of the woman’s social, medical, and obstetric history. In 1996, the Optimality concept was used as the basis for a tool developed to measure the quality of midwifery care in the Netherlands [21]. This work, in turn, was used as the foundation for the Optimality Index-United States (OI-US) [22]. The OI-US has two components: 1) a Perinatal Background index (PBI) – 14 items on pre-existing maternal risk characteristics, such as age, partner status, lifestyle and previous obstetric history and, 2) the Optimality Index (OI) with 42 items on the prenatal, birth and postnatal domains. Optimal care practices are scored with a 1; non-optimal practices are scored as 0. This allows a care professional to calculate a percentage score for both perinatal background factors (PBI) and current pregnancy, birth and postpartum and neonatal items (OI). The OI assumes that a risk free status without medical intervention is the most optimal and scores 100% [23]. The OI-US has been described as a tool in which professionals involved in the provision of maternity care can appraise both processes and outcomes of maternity care for low- and medium risk women [24]. In addition, it may also have potential as an educational tool. A small study (*n* = 9) from the United States examined the OI-US specifically as a tool for learning [25]. The OI-US was used as the basis for a number of educational activities with student midwives. Most students considered it a useful tool for assessing and reflecting on care given during childbirth and for supporting the awareness for evidence-based use of interventions. While these findings are from a small qualitative study and, as such, should be interpreted cautiously, they are of interest to midwifery educators when considering how (future) midwives can best be supported in their role as facilitators of physiological childbirth.

The OI has been validated for use in the United States (OI-US) [23] and, following tailoring to the specific care setting, it has also been validated for use in the United Kingdom [24] and Turkey [26].

Recently, the OI has also been adapted to fit recorded data in a large perinatal database in the Netherlands [27] – this ‘lean’ version of the OI (OI-NL2015) was developed specifically for use in the Dutch Birth Centre Study [28], in which data were used from the Dutch Perinatal Registry (PRN database). The tailoring of the Optimality Index to variables in the PRN database deviates from pre-existing versions of the Optimality Index (OI-US, OI-UK and OI-TR) by including only items available in that database (*n* = 31). While this may be a pragmatic choice aimed at ensuring availability of data, this approach is less robust than a complete adaptation and validation of the Optimality Index specific to a country’s maternity care context, as demonstrated in the United Kingdom and Turkey [24, 26]. Importantly, as Hermus et al. also note, the OI-NL2015 has not been validated for use in the Netherlands [27].

Optimality as a concept is in line with the Dutch perspective of childbirth as a fundamentally physiological process [21]. The evidence-based care practices reflected in its items support non-intervention to enhance physiological childbirth in combination with maximal perinatal outcomes. However, the OI-US is not tailored to care in the Netherlands and has not been validated for use there.

Our study details the development and validation of the OI for the Dutch maternity care setting, creating an Optimality Index-Netherlands (OI-NL) that has face and discriminant validity and is a reliable instrument for measuring application of evidence based care to support healthy physiological childbirth and identifying the care practices that support and promote this physiology.

## Methods

This study was conducted in two phases (a visual overview of these can be found in Appendix). In the first phase, back and forth translation and input for an expert panel (face validity) were used to create a Dutch language version. Subsequently, discriminant validity and inter-rater reliability of the Optimality Index-Netherlands (OI-NL) were assessed.

The study was approved by the ethics committee, Zuyderland Zuyd (16-N-69).

### Development of the OI-NL

Following the steps for cross-cultural validation [29], the OI-US was translated from English to Dutch by two of the authors (ST and MN) both of whom have midwifery expertise in the Dutch setting and are fluent in both languages. Some small discrepancies in translation were resolved by discussion before synthesis into one document. The document was then translated back into English by two linguistic experts and this translation was reviewed for faithfulness to the OI-US version by two of the authors (LKL and RdV), both of whom are familiar with the content and context of the OI-US. Additional file 1 contains an overview of the items contained in both the OI-US and the OI-NL.

### Face validation

A Dutch language translation was then presented to a panel of 10 experts in Dutch maternity care, consisting of community midwives (*n* = 2), hospital midwives (*n* = 2), obstetricians (*n* = 2), midwifery lecturers (*n* = 2) and midwife researchers (*n* = 2). They were requested to review all the items within the OI-NL to determine the relevance and accuracy of the items in relationship to care within the Netherlands. They were also invited to identify items that were not part of the OI-US but were relevant to the Dutch context. The feedback on the PBI (this comprises the first 15 items of the OI-NL) focused on the item pertaining to ethnicity, this was adapted to reflect ethnicity in the Netherlands and simplified for use. Both midwives and obstetricians pointed out the possibility of Body Mass Index (BMI) values being incorrectly scored and suggested collating values for height and weight and calculating the BMI once data was collected in order to reduce errors. The further feedback concerned a number of items which we adapted, it was decided to group previous birth by caesarean section into an item specifying ‘intrapartum complications’, adding ‘instrumental birth’ to the item ‘intrapartum complications’ as they considered these relevant to the obstetric history of multiparous women. They also suggested separating previous fetal death after 16 weeks gestation from previous antenatal complications, as detailed in the OI-US. We also opted to include the item ‘domestic/intimate partner violence’ as a perinatal background factor, adding detail to clarify that this item pertains to both social history and current pregnancy.

For the OI items, the experts suggested the removal of the item ‘non-stress test in pregnancy’ as it is not part of midwife-led antenatal care in the Netherlands. Clarification was requested on the item ‘liquor’ as, without colour classification (clear/meconium stained) it was considered ambiguous. The item ‘delayed cord clamping’ was added, as this is recommended practice in physiological births [30]. We adapted the quantity of blood loss (item postpartum haemorrhage) from 500 to 1000 ml, reflecting the Dutch definition of PPH [31]. A similar adaptation was made for the item on placenta retention, again bringing the duration of time for defining retention in line with Dutch definitions [31]. In addition to gestation at birth, our experts suggested that the index should reflect the certainty of gestation, as first trimester (dating) ultrasonography is a routine part of care in the Netherlands [32]. Following World Health Organization’s (WHO) recommendations for early infant feeding [33], we added early breastfeeding as an item and used the WHO guidelines to define the measure. Further to these suggestions, some minor linguistic feedback was given in order to ensure clarity of wording in the Dutch translation of the OI.

### Pilot test

The OI-NL was then pilot tested in a primary midwifery practice in order to determine its feasibility as a data collection instrument in this environment. Three primary care midwives in one midwifery practice used the OI-NL on 15 occasions. They reported both clarity of items and ease of use.

An English or Dutch language version of the OI-NL is available from the authors.

### Discriminant validity

#### Settings and participants

Between September 2016 and January 2017, 161 Dutch midwifery practices linked to the department of midwifery education at Zuyd University of Applied Sciences were sent written information about this study. They were invited to participate in this study and collect data on the women that started their birth under their care.

#### Data collection

In the midwifery practices that agreed to participate, women were approached for consent either during pregnancy or shortly after birth. Once consent was given, the midwife completed the OI-NL data form in a de-identified manner using information about the pregnancy, labour and birth and early postpartum period recorded in the case notes of each individual client, shortly after each birth. Primiparous and multiparous women who were in midwifery care at the start of labour were included.

#### Power calculation and data analysis

For assessing discriminant validity of the OI-NL, we conducted our data analysis using completed OI-NL forms.

A sample size calculation indicated that 28 completed OI-NLs per defined group (*n* = 56 in total) would be adequate to demonstrate an effect size of 0.5 (α = < 0.05, 80% power) using an independent t-test to compare mean OI scores between two independent groups: primiparous and multiparous women.

For each participant, percentages scores were calculated for the total OI-NL and the PBI and OI part of the OI-NL. These were used for analysis. A priori, we established that forms with more than 10% missing would be excluded. When less than 10% of the items were missing the dominator would be reduced, in accordance with the instructions provided by the OI-US [22].

We assessed discriminant validity by testing two hypotheses. We hypothesized that primiparous women in our data set will demonstrate a lower OI score than multiparous women. This assumption is based on national data, which show that primiparous women have more interventions during birth than multiparous women [17]. This finding will demonstrate that the OI is sensitive to these intervention differences between primiparous and multiparous women. Furthermore, we examined the association between birth-setting and the Optimality Index Score, hypothesizing that women cared for in either a home or out-patient (polyclinic) midwife-led setting will demonstrate significantly higher OI scores than women requiring intrapartum transfer to obstetric-led care [34].

Using SPSS version 24, we conducted multiple linear regressions analysis, using the OI percentage score as dependent variable and parity, PBI score and midwife-led births as independent variables. Parity was a dichotomous variable, coded as 0 for primiparous women and 1 for multiparous women, generating results that indicate the effect of parity, corrected for perinatal background factors. Midwife-led care was a dichotomous variable, coded as 0 for obstetric-led care and 1 for midwife-led care. Significance was set at 5% (two-tailed test).

### Inter-rater reliability

Percentage agreement was measured in order to determine inter-rater reliability between two raters. The first author of this study (ST) and a midwife working with the OI-NL scored a data set from 25 clients in one midwifery practice to examine reliability of the OI-NL as a whole and at item level. Agreement of more than 80% was considered evidence of reliability [35].

## Results

The Optimality Index-Netherlands consisted of a PBI (15 items) and OI (42 items). Discriminant validity and inter-rater reliability were assessed.

### Pilot test

The three midwives, based in a primary care midwifery practice, who tested the OI-NL, reported that the items were clear and easy to interpret. They estimated the time needed to complete the OI-NL at around 10 min and reported that completing the OI-NL in the early postpartum period was the most effective while they were still able to clearly remember details about the client, the care that had been given and while they still had easy access to the medical documentation of each client.

### Confirmation of validity

The 15 midwifery practices that participated in the study were of mixed size, including solo and group practices. The practices were situated throughout the Netherlands covering both urban and rural areas. We sent 505 OI-NL forms to these midwifery practices; of these, 272 completed forms were returned (53.8%). Seventy-six forms were returned with missing items. In 72 cases, missing items constituted no more than 10%. Four forms were excluded as there was greater than 10% of data missing and another two forms were excluded, as these had been completed for women who received obstetric care during pregnancy so were not under the care of the midwife at the start of labour. This left 266 completed forms, more than the a priori 56 forms required for sufficient power sample to assess discriminant validity and sufficient in number to detect differences between the groups, based on the power calculation.

The main characteristics of the participants are presented in Table 1.

**Table 1:** Characteristics of women included in the sample

Age	Participants No. (%)
Mean age	31.05 years
Parity	
Primiparous	95 (35.7%)
Multiparous	171 (64.3%)
Ethnic origin	
Dutch/Western	246 (92.5%)
Non Western	20 (7.5%)
Place of birth	
Home	56 (21.0%)
Hospital – outpatient/polyclinic	59 (22.2%)
Hospital – referred to obstetrician led	81 (30.4%)
Not recorded	70 (26.3%)

### Discriminant validity

The mean PBI percentage scores for primiparous and multiparous women in our study were 92.61 and 91.44% respectively, the mean OI percentage scores for primiparous and multiparous women in our study were 82.98 and 89.00% respectively (Table 2). For women who had midwife-led care, mean PBI scores were 92.66%. For the obstetric-led care, these were 90.02%. Mean OI percentage scores were 90.61% (midwife-led) and 78.54% (obstetric-led) respectively (Table 3).

**Table 2:** Descriptive statistics for parity of the women in our study

	Number	Minimum % score	Maximum % score	Mean % score	Std. Deviation
PBI percentage					
- Total group	266	64.29	100.00	91.88	8.26
- Primiparous women	97	64.29	100.00	92.64	9.39
- Multiparous	169	66.67	100.00	91.44	7.53
OI percentage					
- Total group	266	58.97	100.00	86.81	8.38
- Primiparous women	97	58.97	100.00	82.98	9.80
- Multiparous	169	65.00	100.00	89.00	6.53

**Table 3:** Descriptive statistics for care-setting of the women in our study

	Number	Minimum % score	Maximum % score	Mean % score	Std. Deviation
PBI percentage					
- Total group	196	65.73	100.00	91.57	8.85
- Midwife-led	115	65.73	100.00	92.66	8.38
- Obstetric-led	81	66.67	100.00	90.02	9.31
OI percentage					
- Total group	196	60.20	97.62	85.62	8.42
- Midwife-led	115	76.19	97.62	90.61	4.62
- Obstetric-led	81	60.20	92.86	78.54	7.48

In testing our hypotheses, the multiple linear regressions analysis demonstrates that parity is a significant predictor of the OI percentage score, also when corrected for perinatal factors (PBI score) with a β = 6.21 (*p* = .00) (Table 4).

**Table 4:** Multiple linear regressions: Parity

Model	Unstandardized Coefficients		Standardized Coefficients	t	p	95.0% Confidence Interval for B	
	B	Std. Error	Beta			Lower	Upper
(Constant)	68.38	5.43		12.59	0.00	57.51	79.24
Parity	6.21	0.99	0.36	6.25	0.00	4.23	8.20
PBI percentage	0.16	0.06	0.16	2.72	0.01	0.04	0.27

Furthermore, the professional providing care is a significant factor, with midwife-led care (either at home or out-patient (polyclinic) demonstrating significantly higher OI scores than obstetric-led births (β = 12.1, *p* = .00), when corrected for perinatal factors (Table 5).

**Table 5:** Multiple linear regressions: Care-setting

Model	Unstandardized coefficients		Standardized coefficients	t	p	95.0% confidence interval for B	
	B	Std. Error	Beta			Lower	Upper
(Constant)	79.24	4.46		17.78	0.00	70.33	88.15
Care setting	12.09	0.88	0.71	13.78	0.00	10.34	13.84
PBI percentage	−0.01	0.05	−0.01	−0.16	0.88	−0.11	0.09

#### Inter-rater reliability

Overall rater agreement was calculated to be 98%. We also examined rater agreement per item. For the PBI, rater agreement ranged from 96 to 100%, with just two of the 15 items showing a difference in scoring. Of the 42 items comprising the OI, there were differences in scoring in 16 items, with agreement ranging from 88 to 100%. One item, Apgar score at 5 min, was outside this range, with an agreement rate of 76%. This result suggests that the item Apgar score is sensitive for incorrect scoring when using the OI-NL.

## Discussion

Our study confirms that the OI-NL is a valid and reliable tool for use in the Dutch low risk maternity care setting. We tested the discriminant validity of the instrument by comparing the scores of primiparous and multiparous women and found that the OI-NL was sensitive to differences in obstetric interventions during birth. Primiparous women had significantly lower OI-NL percentage scores than multiparous women, also when corrected for perinatal background factors. Our sample also showed that midwife-led care is associated with lower levels of obstetric intervention. This is line with our hypothesis as in the Dutch primary care settings midwives give care to healthy women with physiological pregnancies and refer women with complications or pathologies for obstetric-led care.

As we noted above, the OI-NL includes one item that is particularly susceptible to rater error. The ‘Apgar Score’ item was the only one with less than 80% rater agreement [34], − perhaps because the clinical record has scores for three different points in time (1, 5, and 10 min). It is likely that this issue can be addressed by emphasizing the use of the 5 min Apgar score in the OI-NL instructions and not the score at 1 or 10 min.

The OI-US has been described as a tool in which professionals involved in the provision of maternity care can appraise both processes and outcomes of maternity care for low- and medium risk women [24]. We consider that the OI-NL could be a useful tool for experienced midwives who may have more routinized care practices or who may be less ‘at home’ with evidence-based ways of working. Many care practices are deeply entrenched in midwifery practice and, as such, it may be difficult for professionals to recognize these – Wagner described this phenomenon metaphorically, using the phrase ‘fish do not know that water exists’, meaning that professional socialisation, particularly where birth is medicalised, can lead to difficulty in pinpointing a ‘normal’ birth [36]. The OI-NL is a tool that may help professionals to ‘see water’, recognize routinized care practices and re-examine and reflect upon these. Evidence-based guidelines that support physiological approaches to childbirth can empower midwives, particularly in medicalized care settings [4]. However, midwives appear to be influenced by factors other than Evidence Based Medicine (EBM), including personal attitudes towards physiological childbirth and inter-professional collaboration with others with similar and differing perceptions of obstetric risk [37]. Midwives also report that in hospital settings, they feel they must ‘account’ for decisions regarding physiological childbirth and experience limitations in the way they can ‘experiment’ with e.g. birthing positions [4]. Providing a validated, evidence-based tool can support midwives in identifying interventions that are evidence based compared to those that are debatable and not evidence-based. While recognizing non-evidence based care is important, other skills, such as the ability to challenge non-evidence based practice and advocate practices that support and promote physiology, irrespective of care setting, will also be relevant. While the evidence is limited to one small study, the OI-NL has potential as an education tool, promoting reflection and discussion about the diversity of midwifery practice that student midwives may experience in midwifery practices in the Netherlands [13] and because it is a valid instrument that captures evidence-based physiological childbirth practices, specific to the Dutch context.

It is perhaps reflective of the dynamism of Dutch midwifery that two studies relating to the Optimality Index in the Netherlands have emerged at the same time [27]. While both of these studies focus on the concept of optimality within the Netherlands, they are fundamentally different, with different purposes. OI-NL2015 was developed for use as a research tool to use within a specific larger study of birth centres. It is linked to the PRN, a large perinatal database, which has both advantages and disadvantages. The link to the PRN allows immediate access to a large amount of data, but it also makes the OI-NL2015 a “lean” instrument, capturing only 31 variables included in the database. The OI-NL is a broader tool with 57 variables, comparable with OI-US [23], OI-UK [24] and OI-TR [26], capturing evidence-based physiological childbirth practices, tailored to the specific Dutch setting. It is valid and offers maternity care providers the opportunity to reflect on both care processes and outcomes from prospective, current data, while data from the PRN database are already 1 to 2 years old at publication. The OI-NL has the additional advantage of being a tool that could be used to evaluate different models of maternity care. The OI-NL2015 was designed to examine maternity care within birth centres. The OI-NL validated here, with the specificity of its 42 items and its attention to variables not available in existing registries, could allow for the assessment and comparison of aspects of the delivery and outcomes of care in a variety of settings. However, an assessment of the effectiveness of the Dutch midwifery care model or comparisons between midwifery and obstetric led models of care were not the focus of this study.

### Strength and weakness of the study

This study has a number of strengths, notably which, as far as we are aware, it is the first validation study of the OI that uses prospective data from midwives offering care for women with physiological pregnancies, rather than pre-existing data sets or clients with mixed risk profiles [26]. While a data set would have offered larger amounts of data for validation, an earlier validation study [24] indicated that the OI indicated potential as a prospective tool but was not tested as such. Our study operationalised this approach, with sufficient prospective data to meet the sample size calculation. This study offers a broad overview of the items relevant for reflection on the physiology of childbirth within the Dutch midwifery care setting, including an assessment of validity.

This study has some weaknesses, namely a large amount of missing data (*n* = 70) for the actual place of birth. The OI-US includes an item on the intended place of birth at the start of the intrapartum period but does not include an item detailing the actual place of birth. In our original translation, we followed the OI-US lead. Reflection and discussion between the authors once data collection had commenced led to a decision, 1 month into data collection, to request that midwives document the care setting (midwife-led home or out-patient (polyclinic) or transfer to obstetric care. While it was useful for us to collate information about the place of birth, it does not affect the assessment of validity of the items contained within the OI-NL.

## Conclusion

Our study confirms that the OI-NL is a valid and reliable instrument that captures the relevant, evidence-based items that support physiological childbirth, tailored to the Dutch maternity care system. Importantly, in a time in which there are concerns about the medicalization of childbirth – in the Netherlands and elsewhere - it brings an evidence-based, physiological approach to childbirth into sharp focus and offers potential as an instrument measuring detailed care processes and outcomes of physiological maternity care in the Dutch System.

### Additional file

Additional file 1: The OI-US and the OI-NL instruments. This file contains the OI-US and the OI-NL presented together so that the reader is able to compare the similarities and differences following the creation and validation of the OI-NL. (DOCX 27 kb)

## Appendix

**Table 6:** Steps in the validation process

Cross-cultural and face validation January – June 2016	
• Back and forth translation by linguistic experts and midwifery professionals • Synthesis into Dutch language document • Input from an expert panel – does the instrument appear to capture the relevant, evidence-based items that support physiological childbirth in the Netherlands • Pilot test	
Assessment of discriminant validity and inter-rater reliability September 2016 – March 2017	
• Collect data in primary midwifery practices using the OI-NL instrument • OI-NL forms returned from primary care midwives (*n* = 266) • Assessment of discriminant validity - Differences in OI-NL percentage score between primiparous and multiparous women - Differences in OI-NL percentage score between midwife-led care births and births referred to obstetric care • Inter-rater reliability	

This table contains an overview of steps taken in the validation process and the time frame in which this process occurred

## Abbreviations

EBM: Evidence Based Medicine
OI: Optimality Index
OI-NL: Optimality Index-Netherlands
OINL-2015: Optimality Index Netherlands-2015
OI-TR: Optimality Index Turkey
OI-UK: Optimality Index United Kingdom
OI-US: Optimality Index-United States
PBI: Perinatal Background Index
PPH: Postpartum Haemorrhage
PRN: Perinatal Registry Netherlands
SPSS: Statistical Package for the Social Sciences
WHO: World Health Organization
