# Supplementary material for: Creating an Optimality Index – Netherlands: a validation study
Source: BMC Pregnancy Childbirth. 2018 Apr 16;18:100. doi: 10.1186/s12884-018-1735-z (PMC5902845; doi:10.1186/s12884-018-1735-z)
Supplement: Supplementary file 1 — The OI-US and the OI-NL instruments. This file contains the OI-US and the OI-NL presented together so that the reader is able to compare the similarities and differences following the creation and validation of the OI-NL. (DOCX 27 kb) [file 12884_2018_1735_MOESM1_ESM.docx]

Additional file 1

The Optimality Index-US and the Optimality Index-NL.

The cross-cultural and face validation process is described in the method and results section of this paper. Areas that were adjusted to the Dutch situation have been highlighted.

The NL-OI adapted some sequential changes in order to make the index as easy to use as possible.

| Optimality Index-US |  | Optimality Index-NL |
| --- | --- | --- |
| *Perinatal background Index* |  | *Perinatal background Index* |
| 1. Marital status, as if married |  | 1. Married or cohabiting |
| 2. Ethnic minority (non-minority) |  | 2. European or Western ethnicity |
| 3. Smoking |  | 3. Smoking (since conception) |
| 4. Alcohol |  | 4. Alcohol use (since conception) |
| 5. Drug use |  | 5. Drugs use (since conception) |
| 6. Pre-pregnancy body mass index weight (in KG)/height (in M) |  | 6. Height and weight  (BMI) |
| 7. age |  | 7. Age |
| 8. Pre-existing major, chronic disease   - Hypertension - Chronic renal disease - Diabetes (nongestational) - Heart disease class II-IV - HIV antibody positive - major psychiatric history (treated with drugs or inpatient therapy) |  | 8. Pre-existing serious chronic diseases   - Hypertension - Chronic renal disease - Diabetes Mellitus - Heart disease type II-IV (shortness of breath in absence of exertion) - HIV antibody positive - Serious psychiatric illness (requiring medication or admission to hospital) |
| 9. inter-pregnancy interval between index pregnancy and previous viable birth > 18 months and < 60 months |  | 9. Pregnancy interval of this (index) pregnancy and previous birth > 18 months and < 60 months |
| 10. previous preterm delivery < 37 weeks |  | 11. Previous preterm delivery <37 weeks gestation |
| 11. previous intrauterine fetal death |  | 13. Previous history of fetal death >16 weeks |
| 12. previous Cesarean section |  | 12. Previous intrapartum history of   - Postpartum Haemorrhage - Retained placenta - Instrumental delivery - Caesarean Section |
| 13. previous baby < 5 1/2 pounds at birth |  | 14. Previous history of baby with birth weight < 2500 gram |
| 14. other serious antepartum complications (history of)   - gestational diabetes - intrauterine fetal demise - placenta praevia - preeclampsia - eclampsia - pyelonephritis - Rhesus antagonism - Vaginal bleeding in 2nd or 3rd trimester, from cause other than placenta praevia |  | 10. Previous antenatal history of   - Diabetes Gravidarum - Placenta Praevia - Pre-eclampsia - Eclampsia - Pyelonephritis - Rhesus antagonism - Vaginal blood loss in 2^nd^ or 3^rd^ trimester (excluding placenta praevia) |
| *Optimality index* |  |  |
| *Present pregnancy, diagnostic and therapeutic measures* |  |  |
| 15. intrauterine fetal demise |  |  |
| 16. domestic violence  (includes intimate partner) |  | 15. Previous or current domestic violence, intimate partner violence or sexual violence |
|  |  | *Optimality index* |
| 17. other serious antepartum complications (current pregnancy)   - anemia (Hgb < 10 gm in any trimester) – not improved with treatment - diabetes diagnosed in pregnancy - major psychiatric history (formally diagnosed or treated with drugs/inpatient therapy) - multiple birth (twins or higher number of births anticipated) - placenta praevia - pre-eclampsia (diagnosed in antepartum period) - pyelonephritis - Rh sensitization - Vaginal bleeding in 2nd or 3rd trimester, from cause other than placenta praevia. |  | 16. In this pregnancy   - Anaemia (<5.6mmol in each trimester, non-responsive to therapy) - Diabetes Gravidarum - Psychiatric illness (officially diagnosed and requiring drug therapy and/or admission to hospital) - Multiple pregnancy - Placenta previa - Pre-eclampsia - Pyelonephritis - Rhesus antagonism - Vaginal blood loss in 2^nd^ or 3^rd^ trimester (excluding placenta praevia) |
| 18. prenatal care: initiation in first trimester (<14 weeks) and minimum of 5 visits |  | 17. Antenatal care initiated in first trimester (<14 weeks) and consisting of at least five consultations |
| 19. amniocentesis  NOTE: that if CVS is documented, a note should be added to the abstraction record |  | 18. Invasive prenatal diagnostic testing |
| 20. nonstress test/contraction stress test/biophysical profile |  |  |
| 21. medication use |  | 19. Medication use (pregnancy specific) |
| 22. period of time between first digital examination following rupture of membranes and birth |  | 20. Time interval between first vaginal examination following rupture of membranes and birth is <24 hours |
| 23. amniotic fluid |  | 21. Clear liqour |
| 24. induction/augmentation of labor |  | 22. Induction/augmentation of labour |
| 25. amniotomy |  | 23. Artificial rupture of membranes |
| 26. oral or injectable (IM or IV) medication during first or second stage of labor |  | 24. Medication (oral, IM or IV injection) during first or second phase of labour (including analgesia) |
| 27. epidural analgesia for labor and/or birth |  | 25. Epidural anaesthesia during labour |
| 28. fetoscope, Doppler, or intermittent electronic monitoring during labor (rather than continuous electronic fetal monitoring) |  | 26. Intermittent auscultation of the foetal heart using a Doppler fetal monitor or CTG (not continuous fetal monitoring) |
| 29. fetal heart rate abnormalities that altered management of the labor process |  | 28. Fetal heart pathology leading to change in treatment/management during labour |
| 30. presence of a support person during labor (other than care provider) |  | 27. Presence of someone that supports the woman during labour (other than a healthcare professional) |
| 31. nondirected pushing |  | 29. Spontaneous bearing down (without rigid pushing instructions) |
| 32. delivery occurred in the place originally intended at the onset of labor |  | 30. Birth took place at location planned at start of labour |
| 33. nonsupine position at birth |  | 31. Non-supine birthing position during second stage and/or at the time of birth. |
| 34. presentation at birth |  | 32. (Cephalic) presentation at birth |
| 35. instrumental (vaginal) delivery |  | 33. Instrumental birth (vaginal) |
| 36. Cesarean section |  | 34. Caesarean Section |
| 37. episiotomy |  | 35. Episiotomy |
| 38. 1st or 2nd degree laceration of perineum or perineal tissue requiring sutures (including sulcus and cervical lacerations) |  | 36. 1st or 2nd degree rupture or other perineal/genital trauma requiring sutures |
| 39. 3rd or 4th degree extension of either an episiotomy or a 1st or 2nd degree laceration |  | 37. 3rd or 4th degree perineal rupture |
| 40. medication (other than oxytocin or local anesthetic for perineal repair) during the third stage of labor |  | 38. Medication (other than Oxytocin or regional anaesthesia for suturing purposes) during the 3^rd^ stage of labour |
| 41. skin-to-skin contact |  | 39. Skin to skin contact between mother and baby directly after birth |
|  |  | 40. Delayed cord clamping |
| 42. placental retention (≥ 30 minutes) |  | 41. Retained placenta (>60 minutes) |
| 43. postpartum hemorrhage (provider’s documentation that this did not occur; actual amount of blood loss not relevant) |  | 42. Postpartum blood loss >1000 ml (as estimated by care giver) |
| 44. blood transfusion |  | 43. Blood transfusion |
| 45. other serious intrapartum complications   - chorioamnionitis - cord prolapse - eclampsia - placental abruption - pre-eclampsia present during intrapartum period - shoulder dystocia |  | 44. Complications during the intrapartum period   - (suspected) chorioamnionitis - Cord prolapse - Pre-eclampsia - Eclampsia - Placental abruption - Shoulder dystocia |
| 46. estimate of gestational age |  | 45. Gestation at birth (weeks and days) |
|  |  | 46. Certainty of gestational age |
| 47. birth weight |  | 47. Birth weight |
| 48. Apgar score at 5 minutes |  | 48. Apgar score at 5 minutes |
| 49. transfer to high risk neonatal care setting |  | 49. Admission/transfer to neonatal unit |
| 50. congenital anomalies |  | 50. Congenital abnormalities |
| 51. birth trauma, or other serious medical problem   - bacterial infections other than sepsis - bronchopulmonary dysplasia - cardiac failure - hypovolemia, hypotension, shock - intraventricular hemorrhage - necrotizing enterocolitis - pneumonia - persistent pulmonary hypertension - renal failure - respiratory distress syndrome - Rh disease - Sepsis |  | 51. Birth trauma or other serious medical problems   - sepsis - other bacterial infections - bronchopulmonary dysplasia - cardiac failure - hypovolaemia, hypotension, shock - intraventricular haemorrhage - necrotizing enterocolitis - pneumonia - persisting pulmonary hypertension - renal failure - respiratory distress syndrome - haemolytic disorders/hyperbilirubinia |
| 52. breastfeeding  (time period: at time of mother’s discharge from birth setting or up to 72 hours postpartum) |  | 52. Breastfeeding initiated within 2 hours of birth |
|  |  | 53. Breastfeeding at 72 hours postpartum |
| 53. perinatal death:  (time period birth: up to 72 hours of age) |  | 54. Perinatal death within 72 hours |
| 54. fever (100.4 degrees F or higher) while mother remains in the birth setting, OR provider diagnosis of infectious process or major complication   - cystitis - endometritis - hematoma - local infection of sutures - mastitis |  | 55. Maternal pyrexia (>38^o^C at 72 hours pp) or other complication   - cystitis - endometriosis - haematoma - infected suture site - mastitis - secondary postpartum haemorrhage - thrombosis/emboli |
| 55. prescription medications for conditions newly identified in IP or PP period (exception: iron and vitamins, oral contraceptives, RhoGam©, rubella vaccine) |  | 56. Medication prescribed for new conditions diagnosed during labour or in the post-natal period (exceptions: iron and vitamins, oral contraceptives, Anti D immunoglobulins, Rubella vaccine) |
| 56. maternal mortality |  | 57. Maternal death |
